# The impact of methylene blue in colon cancer: a retrospective multicentric study

**DOI:** 10.1007/s00384-024-04663-2

**Published:** 2024-06-13

**Authors:** Alexandre Carvalho, Manuel Limbert, Francisco Cabral, Ana Fareleira, Alexandre Duarte, Rita Barroca, André Goulart, Pedro Leão

**Affiliations:** 1 Life and Health Sciences Research Institute (ICVS), Medical School, University of Minho, Braga, Portugal; 2 Instituto Português de Oncologia de Lisboa, Lisbon, Portugal; 3 Hospital S. João, Porto, Portugal; 4 Hospital Lusíadas, Lisbon, Portugal; 5 General Surgery Department, Grupo Trofa Saúde, Braga, Portugal; 6 ICVS/3B’s - PT Government Associate Laboratory, /Guimaraes, Braga, Portugal

**Keywords:** Colon cancer, Lymph node harvest, Intra-arterial methylene blue

## Abstract

**Introduction:**

Discussions about the optimal lymph node (LN) count and its therapeutic consequences have persisted over time. The final LN count in colorectal tissues is affected by a variety of variables (patient, tumor, operation, pathologist, immune response). Methylene blue (MB) intra-arterial injection is a simple and inexpensive procedure that can be used to enhance lymph node count.

**Aim:**

Analyze whether there is a statistically significant difference between intra-arterial methylene blue injection and conventional dissection for the quantification of lymph nodes and determine if there is a variation in the quality of lymph node acquisition.

**Methods and results:**

Between 2015 and 2022, we conducted a retrospective analysis of colon cancer specimens. Data on the tumor’s features, the number of lymph nodes, the number of lymph nodes that were positive, and other factors had been collected. The number of identified lymph nodes was highly significantly improved in the study group (*P* < 0.05). There is not a significant statistical difference between groups regarding the metastatic lymph node harvest. The group with injection of intra-arterial methylene blue shows a significantly decreased (*P* < 0.05) of the of cases with less than 12 lymph nodes recovered comparing with the control group.

**Conclusion:**

Colon cancer specimens can be easily evaluated concerning lymph nodes using the methylene blue method. Therefore, we strongly advise this approach as a standard procedure in the histological evaluation of colon cancer specimens in order to maximize the identification of lymph nodes. However, the detection of metastatic lymph nodes was unaffected significantly.

**Supplementary Information:**

The online version contains supplementary material available at 10.1007/s00384-024-04663-2.

## Introduction

The main determinant of whether adjuvant chemotherapy should be given to patients with colorectal cancer (CRC) is the result of the histological lymph node (LN) examination [1]. Adjuvant therapy dramatically reduces mortality and the chance of recurrence in stage III (T1–4, N1-2, M0) colorectal cancer when compared to surgery alone [2]. When LN metastases are evident, stage III tumors should be treated with adjuvant chemotherapy [3]. Actually, the prognosis for patients with metastatic LNs was worse than that of those without them [4]. In stages I and II, the 5-year survival rates vary from 82 to 93%, while in stage III (lymph node metastases), the rates drop to 59% [5]. Furthermore, 20% of stage II cancers exhibit a clinical history that is surprisingly aggressive, and adjuvant therapy, which is well acknowledged, is beneficial for these individuals [1]. Patients who have undergone planned curative surgery may experience up to 30% relapse with local and/or distant metastases, depending on the stage of the malignancy [6]. Given the high likelihood of recurrence, accurate and thorough lymph node examination is crucial for histopathological staging, which in turn affects prognosis estimation and therapy stratification in colorectal cancer [6].

The precise assessment of the lymph node condition is one of the most important factors in this scenario. For an accurate assessment of lymph node status in colorectal cancer resection specimens, the UICC (International Union Against Cancer) recommends examining a minimum of 12 lymph nodes, though previous recommendations have varied significantly in a range from 9 to 18 lymph nodes [3, 7]. Despite these recommendations, it has been reported that this required number of LNs has not been detected or analyzed in several colorectal tissues. Among patients with colon cancer, neoadjuvant radiochemotherapy has a particularly detrimental effect on the harvest. Understaging in colorectal cancer is thought to result from a failure to gather lymph nodes for pathological analysis [2].

Histopathologists traditionally rely on manual palpation to examine surgical specimens. However, this method may overlook small nodes, and it is recognized that nodes with a diameter of less than 5 mm could constitute up to half of all metastatic nodes [8]. To address this limitation, numerous approaches have been devised to enhance the dissection and analysis of a greater number of lymph nodes in colorectal cancer (CRC) surgical specimens, thereby improving lymph node staging [9]. Techniques such as compression procedures, methylene blue-assisted lymph node dissection (MBLND), and fat-clearing protocols have shown promise in increasing lymph node detectability. Nonetheless, these methods are time-consuming, costly, and may involve potentially hazardous substances [10].

This study holds significance as, thus far, no retrospective multicenter study has compared the effectiveness of MBLND to the conventional method in patients with colon cancer. The findings of this research have the potential to influence the approach taken by pathologists in managing patients with colon cancer.

## Material and methods

### Study

This is an observational study (retrospective cohort), analytical and longitudinal, whose sample focuses on patients undergoing colorectal surgery.

### Patients

Patients were recruited from three medical institutions in Portugal: the General Surgery Department at Grupo Trofa Saúde, Braga (44 participants); Hospital S. João, Porto (37 participants); and Portuguese Institute of Oncology (IPO), Lisbon (74 participants), spanning the period from January 2015 to December 2022. A total of 300 patients were initially considered, with 155 ultimately included in the retrospective multicenter study. Inclusion criteria encompassed both males and females above 18 years of age scheduled for laparoscopic surgery due to colon cancer. The study focused on oncologically resected colon specimens with confirmed or suspected cancer, including node-positive colon cancer cases with or without neoadjuvant therapy. Additionally, patients classified as pN1c according to the seventh edition of the UICC TNM classification, and those undergoing curative resection for histologically proven adenocarcinoma of any colon segment, were included. Exclusion criteria comprised palliative or emergency resections, positive resection margins, death within two months post-operation, recurrent or metastatic colon cancer, and cases involving intestinal obstruction, perforation, or locoregional recurrence surgery and neoadjuvant chemoradiotherapy.

Standard lymph node dissection procedures did not involve staining with methylene blue. Both stained and non-stained colon specimens underwent examination at the Department of Pathology following 24 h of formalin fixation. Lymph nodes were identified through visual inspection and manual palpation of the mesocolon, followed by microscopic examination for metastatic involvement. Tumor staging and classification utilized the TNM system of the American Joint Committee on Cancer. Table 1 outlines the study and control group characteristics, tumor histologic type, surgery type, approach, TNM staging, and operative piece length.

**Table 1:** Patient group characteristics

	Methylene blue — stained	Unstained
Mean ± SD age (y)	69.07 ± 11.22	68.94 ± 11.53
Sex		
Male	43	34
Female	30	38
Histology		
Adenocarcinoma	73	72
Surgery		
Right colectomy	48	42
Left colectomy	12	11
Sigmoidectomy	13	9
Approach		
Laparatomy	24	15
Laparoscopy	49	57
pT		
1	6	7
2	10	9
3	35	35
4	12	12
Not evaluated	10	9
Operative part length ± SD (cm)	23.49 ± 10.60	26.78 ± 13.55

For tumor staging and case classification, we used the TNM system of the American Joint Committee on Cancer. Table 1 presents the characteristics of the study and control group, tumor histologic type, type of surgery, the surgery approach, TNM staging and the operative piece lengh.

### Advanced lymph node dissection

Advanced lymph node dissection techniques involved identifying the main artery (ileocolic, middle colic, or inferior mesenteric artery) and cutting the clip or ligature. The artery was longitudinally opened to facilitate cannulation with a standard 16- or 17-gauge intravenous catheter, without a steel mandarin. To secure the catheter in the artery, a clamp was affixed parallel to the artery. Successful injection of 15 to 20 mL of methylene blue solution (50 mg diluted with 0.9% saline; ratio 1:3) was confirmed by instantaneous blue staining of the specimen’s serosal layer.

### Statistical analysis

The main focus of the study was on two primary outcomes: the total count of lymph nodes and the rate of identifying 12 or more lymph nodes. Additionally, the study considered a secondary outcome measure: the count of metastatic lymph nodes.

To analyze the data, the mean and standard deviation were employed to determine the number of lymph nodes in each group. Correlation between means was assessed using either a two-tailed *t*-test or the Mann–Whitney *U* test, depending on the normality of the results. A significance threshold of <0.05 was adopted to determine statistical significance.

## Results

### Patient population

The study included 73 patients in the MBI group and 72 patients in the control group. Patient characteristics are outlined in Table 1. No statistically significant differences (*p* < 0.05) were observed between the methylene group (*n* = 73) and the control group (*n* = 72) regarding gender, age, distribution of pT stage, size of the operative site, and the proportion of non-laparoscopic versus laparoscopic surgeries.

### Lymph nodes harvest

In the study group, the mean number of harvested lymph nodes (Table 2) was 26.79 ± 13.33, whereas in the control group, it was 21.75 ± 15.64. This disparity was found to be statistically significant (*P* = 0.04, Mann–Whitney test).

**Table 2:** Lymph node harvest

	Methylene blue — stained	Unstained	*p*
Mean ± SD LN number	26.79 ± 13.33	21.75 ± 15.64	*p* < 0.05
Mean ± SD metastatic LN number	0.95 ± 2	1.16 ± 2.351	*p* > 0.05

We further investigated the presence of lymph nodes with metastases in each group (Table 2). The mean number of metastatic lymph nodes was 0.95 ± 2 in the study group and 1.16 ± 2.351 in the control group. However, this difference was not statistically significant (*p* = 0.562, Mann–Whitney test).

Once three different hospital centers were integrated and, therefore, with different pathology groups, the means of the lymph nodes quantified in each of the groups were compared (Table 3). After the statistical analysis, it was found that there was no statistically significant difference (*p* <0.05), regarding the quantification of lymph nodes, between the pathological anatomy services of the different hospitals that integrated the study both in the group of samples subjected to staining by intra-arterial injection of methylene blue and in those that belonged to the control group.

**Table 3:** Lymph node harvest between hospitals

	Hospital Trofa Saúde	Hospital S.João	IPO Lisboa	*p*
Mean ± SD LN number				
Methylene Blue — stained	29.46 ± 16.730	23.37 ± 10.073	26.42 ± 10.937	< 0.001
Unstained	20.63 ± 14.997	29.72 ± 21.548	18.26 ± 10.606	<0.001

Instances of insufficient node harvest, defined as <12 identified lymph nodes, were observed in 6 cases in the study group and in 13 cases in the control group (Table 4). According to the American Joint Committee on Cancer guidelines, the discrepancy between an adequate and an inadequate harvest across the two groups was statistically significant (*p* < 0.05, Fisher’s exact test).

**Table 4:** Optimal lymph node harvest

	Methylene blue — stained	Unstained
Specimens with <12 LN	6	13
Specimens with >12 LN	67	59

## Discussion

The presence of lymph nodes in colorectal cancer patients plays a pivotal role in tumor staging classification, postoperative treatment decisions, and overall prognosis. Among the prognostic factors, the number of detected lymph nodes stands out as particularly significant. As per the American Joint Committee on Cancer guidelines, the detection of at least 12 lymph nodes is recommended for a more accurate diagnosis. Various factors, including age, tumor location, obesity, immune response, neoadjuvant therapy, surgical technique, and effective dissection methods, can influence the quality of lymph node yield. Insufficient identification and examination of lymph nodes have been identified as a primary cause of understaging in colorectal cancer cases.

Among the several available techniques for pathological analysis of colorectal specimens, methylene blue staining was selected for its cost-effectiveness and simplicity. This technique does not necessitate advanced equipment or extensive knowledge. Methylene blue, containing formalin, aids in the fixation process, potentially enhancing specimen fixation. Injecting a small volume of methylene blue solution per specimen stains the lymph nodes a clear blue color, making them distinctly visible against the light bluish intestinal fatty tissue. This facilitates easier and quicker identification of both large and small lymph nodes.

Our study utilized a retrospective design with study and control groups comprising all colon cancer specimens received by three Portuguese hospitals between 2015 and 2022. Analysis revealed no significant differences between the two groups regarding sex, age, surgery location, pT stage classification, operative length, and type of surgery (laparoscopic or open).

Results from our study unequivocally demonstrate a substantial improvement in lymph node identification using intra-arterial methylene blue compared to classical dissection, with a statistically significant difference observed between the study and control groups (26.79 ± 13.33 vs. 21.75 ± 15.64).

However, our study did not observe a significant statistical difference in the number of metastatic lymph nodes recovered after methylene blue application compared to the control group. Consistent with other studies, although methylene blue has been shown to increase the number of harvested lymph nodes, it does not appear to enhance the detection of nodes with metastases. While some studies suggest an increase in metastatic lymph node detection with methylene blue, our multicentric retrospective study aligns with findings indicating no significant difference between methylene blue and classical dissection in terms of metastatic lymph node detection.

Furthermore, our study highlights that methylene blue injection reduces the number of cases where pathologists fail to detect more than 12 lymph nodes (6 cases in the study group vs. 13 cases in the control group), with a significant statistical difference between the groups (*p* < 0.05). This supports the notion from previous studies that methylene blue may offer advantages over classical dissection, as achieving a yield of more than 12 lymph nodes optimizes staging accuracy in colorectal disease.

## Conclusion

We assert that the methylene blue technique proves highly effective in streamlining lymph node assessment in colon cancer specimens. Therefore, we strongly advocate for the incorporation of this procedure as a routine practice in the histopathological examination of colon cancer specimens, aiming to optimize and expedite lymph node identification. However, there was no significative influence in the detection of metastatic lymph nodes.

## Supplementary information

ESM 1 (PDF 99 kb)

## Data Availability

All materials are available for review upon request.
